# Coping strategies and post traumatic stress injury symptoms differ between tactical and frontline police officers: implications for health and public safety

**DOI:** 10.3389/fpsyg.2026.1799457

**Published:** 2026-05-28

**Authors:** Patrick Fahim, Amanda Jani, Loridee De Villa, Judith P. Andersen

**Affiliations:** 1 Department of Psychological and Brain Sciences, University of Toronto at Mississauga, Mississauga, ON, Canada; 2 Affiliated Faculty, Temerty Faculty of Medicine, University of Toronto, Toronto, ON, Canada

**Keywords:** alcohol use, coping, law enforcement, police, post-traumatic stress, PTSD, PTSI, public safety personnel

## Abstract

**Introduction:**

Public safety personnel operate under acute threat and limited control, with elevated rates of post-traumatic stress symptoms across occupations. Maintaining their health and functional capacity is essential for individual wellbeing and effective service delivery. However, much of the literature treats these personnel, including police, as a homogeneous group, limiting understanding of role-specific risk processes. Emerging evidence indicates that different roles involve distinct stressors, organizational contexts, and coping demands. The present study examined role-specific differences in post-traumatic stress symptoms and coping strategies among frontline and tactical police officers to clarify mechanisms of distress and inform targeted interventions.

**Methods:**

Operational stress, coping strategies, PTSD symptomatology, and alcohol use were assessed via self-report questionnaires among 178 (*F* = 24) active-duty police officers, including Canadian frontline officers (*n* = 124; *m*_age_ = 32.5) and tactical officers (*n* = 54; *m*_age_ = 34) from Finland (*n* = 44) and Canada (*n* = 10). This study reports an analysis of original data drawn from prior police studies in the Hart Lab Police Database (HLPD) that use a shared methodology and permit sex-based analyses.

**Results:**

Frontline officers reported significantly higher PTSD symptoms and operational stress than tactical officers (*p* < 0.001 for both). Tactical officers reported higher anxiety symptoms with a small effect (*p* < 0.001). No sex-based differences and no group differences in alcohol use were observed. Coping strategies differed by role with a medium effect (*p* < 0.001). *Post hoc* analyses indicated that tactical officers reported greater use of acceptance, emotional and instrumental support, planning, venting, humor, positive reframing, and self-blame.

**Conclusion:**

Frontline officers reported higher psychological and operational stress than tactical officers, despite tactical roles being defined by deployment to the highest risk calls. Frontline officers did not endorse greater use of coping strategies, suggesting reduced coping variability. Limited flexibility in matching coping responses to high intensity, low controllability stressors may contribute to elevated distress in frontline roles. These findings highlight the importance of examining occupational subgroups within policing to inform role specific prevention and health intervention strategies.

## Introduction

Public safety personnel (PSP) is a broad category that includes (but is not limited to); correctional officers, paramedics, dispatchers, firefighters, and police officers. Individuals in these occupations are routinely exposed to potentially traumatic incidents and experience high levels of occupational stress (Ricciardelli et al., 2020; Carleton et al., 2019). Occupational stress can be divided into operational and organizational stressors. Operational stressors arise from responsibilities related to the job (Carleton et al., 2020), including exposure to potentially dangerous situations in policing. In contrast, organizational stressors arise from the workplace setting itself (Carleton et al., 2020), such as interpersonal conflict with other team members or supervisors. The organizational context in policing plays a major role in mental well-being and help-seeking behaviors, as there is substantial stigma associated with seeking help within police culture (Newell et al., 2021). Various studies surrounding the mental wellbeing of Canadian PSP in recent years have yielded concerning conclusions. An influential paper by Carleton et al. (2018) found that Canadian PSP had a higher rate of positive screens for mental disorders than international PSP (10–35%) and a much higher rate (44.5%) as compared to the general population (10.1%). As a heterogenous group, there exists relevant differences within the PSP category itself. For example, in the same analysis by Carleton et al. (2018), municipal/provincial police and firefighters were found to have the lowest rates of positive screens for mental disorders compared to other PSP categories, albeit still typically higher than the general population. Consequently, scholars have emphasized the need to examine PSP groups separately to identify specific risk and resilience factors (Carleton et al., 2018; Krakauer et al., 2020; Tessier and Genest, 2025; Klimley et al., 2018). In-depth analyses of specific PSP subgroups may also help inform practice recommendations for the improvement of wellbeing and occupational performance.

Police officers are a PSP subgroup who warrant attention due to their high potential for occupational stress and burnout, which may have implications for public safety (Ugwu and Idemudia, 2024). A systematic review by Wagner et al. (2020) reported that the type and frequency of exposure to critical incidents, as well as comorbid mental health disorders, consistently predict elevated risk of post-traumatic stress disorder (PTSD) among police officers. According to a systematic review and meta-analysis, one in every seven police officers experience PTSD or depression (Syed et al., 2020). Police develop disproportionate rates of post-traumatic stress injuries (PTSIs) associated with high stress occupational duties. PTSI is an umbrella term that refers to mental health conditions, including anxiety, depression, post-traumatic stress disorder (PTSD), and substance abuse (Carleton et al., 2018; Violanti et al., 2017; Komarovskaya et al., 2011). Systematic analyses reveal that as many as one in 10 police officers may show symptoms for an anxiety disorder or suicidal ideation (Syed et al., 2020).

A broad literature exists demonstrating sex differences in mental health symptoms. Males more frequently exhibit externalizing problems, characterized by outward-directed behaviors such as aggression, impulsivity, and conduct-related difficulties. In contrast, females more commonly exhibit internalizing problems, characterized by inward-directed distress including anxiety, depression, and social withdrawal (Farhane-Medina et al., 2022). These internalizing patterns overlap substantially with the symptomatology of post traumatic stress injury (PTSI), particularly with respect to anxiety and depressive features.

Consistent with broader mental health literature, sex differences are observed in PTSI among police officers, though findings are not uniform. Several studies in Canada and internationally report a higher prevalence of mental health concerns and PTSI symptoms among female officers based on self-report measures (Angehrn et al., 2022; Carleton et al., 2018; Syed et al., 2020; Van der Meer et al., 2017). In contrast, a large Canadian study (*n* = 1,348) found that female officers were more likely to report anxiety but less likely to screen positive for PTSD (Carleton et al., 2024). This divergence suggests that symptom checklists alone may not capture relevant mechanisms. For example, Beagley et al. (2018) identified empathy as a moderator of post traumatic stress and depressive symptoms in women, indicating that emotional and contextual factors may shape symptom expression. Consistent with this, female officers also report higher levels of organizational and operational stress (Acquadro Maran et al., 2015). Together, these findings highlight heterogeneity in sex differences and suggest that factors such as empathy, stress exposure, and internalizing tendencies may contribute to observed patterns of PTSI. Accordingly, given these inconsistent findings and unresolved questions, examining sex differences remains necessary in police research.

It is also important to acknowledge that specific roles within policing may affect health in distinct ways. In Canada, as in other Western countries, a major distinction within policing is between frontline and tactical officers. Frontline officers complete basic training and perform duties such as traffic stops, 911 responses, and neighborhood patrols. Tactical officers, on the other hand, undergo specialized training, compete for selection, maintain demanding fitness standards, use advanced equipment, and respond to extreme emergencies [e.g., hostage situations, kidnapping; Towns and Ricciardelli, 2025; Toronto Police Service, n.d.; Finnish Police (n.d.)]. However, a study from Planche et al. (2019) suggests that differences between these two groups extend beyond training, manifesting in distinct physiological and psychological profiles. For example, they found that tactical officers display a significantly greater salivary diurnal cortisol response compared to frontline officers (Planche et al., 2019). Counterintuitively, the same study also found that tactical officers reported less operational stress (stress resulting from their policing duties) than their frontline counterparts (Planche et al., 2019). But it is unclear whether these differences reflect pre-existing traits that influence individuals’ attraction to specific roles, or whether they are shaped by role-specific demands. As per the latter, Planche et al. (2019) mentioned the possibility that team cohesion in tactical units, as a function of occupational requirements (Towns and Ricciardelli, 2025) may positively influence their perception of stress. Another remaining unknown is whether frontline and tactical officers differ in their experience of PTSI symptoms, given their distinct occupational exposures, and the critical role that coping mechanisms play in how individuals manage occupational stress.

Coping strategies encompass the behavioral and psychological efforts to meet the demands of stress. Measures of coping, such as the Brief ‘Coping Orientation to Problems Experienced’ (Brief COPE) scale, are based on a definition of coping proposed by Lazarus (1966). Lazarus (1966) conceptualizes coping as an initial appraisal of perceiving a threat, a secondary appraisal recalling a potential response after evaluating available resources and options for coping, and finally, the actual act of engaging in that response. However, the likelihood of distress increases when available resources are perceived as insufficient for meeting situational demands. Although coping strategies ought not be considered through a simplistic dichotomous lens, such as, adaptive vs. maladaptive coping (it is prudent to consider a range of factors such as the context of the individual), police literature often attempts to use this dichotomy to describe behaviors that are not conducive to optimal functioning in the context of policing. For example, strategies such as positive reframing are considered adaptive for police while strategies such as avoidance are considered maladaptive (Modula et al., 2024). A study of Italian police officers found that most officers engaged in seemingly maladaptive strategies such as overactivation (i.e., anger, rumination, feeling overwhelmed) and deactivation (disengagement, suppression of emotion; Civilotti et al., 2021). The same study also found that these strategies were associated with increased experiences of depression, emotional exhaustion and depersonalization (Civilotti et al., 2021). A review by Modula et al. (2024) examined the coping strategies of police from a wide range of countries from 1983 to 2022. While they identified a variety of adaptive and maladaptive strategies, they did not divide officers by occupational role. Confrontation, problem-solving, positive reappraisal, and spirituality were among the adaptive strategies identified; they have been shown to allow officers to maintain a positive outlook and better regulate their emotions in the face of on-going stress (Modula et al., 2024). Avoidance, self-distraction, and social isolation were among the maladaptive coping strategies identified; they are associated with depression, stress, and suicidal ideation among police (Modula et al., 2024). Pitel et al. (2021) demonstrated that Canadian officers who attempted to cope with trauma on their own (or avoided it) were more likely to display impairment in home, work, and relational domains. Research has shown sex and gender differences in coping strategies among police, with women typically reporting greater use of adaptive coping mechanisms than men. For instance, Bonner and Brimhall (2021) found that American female officers utilized more of the following coping strategies than their male counterparts: active coping, planning, religion, positive reframing, emotional support, and instrumental support. In line with this, Chu (2024) examined sex differences in coping strategies and well-being during the Covid-19 pandemic among Taiwanese police officers and found that male officers were more likely to engage in avoidance coping, whereas female officers were more likely to adopt emotion regulation strategies. Alcohol use is also considered a maladaptive coping strategy in the context of policing and beyond, with evidence of negative overall outcomes. Research has demonstrated that, in policing contexts, problematic drinking is associated with heightened PTSI symptoms and specifically work-related traumatic stress (Baker et al., 2022; Irizar et al., 2021; Chopko et al., 2013). Moreover, a study by Ménard and Arter (2013) showed a link between officers’ negative and avoidant coping strategies and increased problematic alcohol use and PTSD symptomology. However, males were more likely to report problematic alcohol use than their female counterparts (Ménard and Arter, 2013). Moreover, a systematic review by Krishnan et al. (2022) found substance misuse (alcohol and drugs) in police offices as being directly linked to completed suicide in 40% of their included studies.

The current study extends prior research by examining differences in police roles (frontline vs. tactical) across PTSI symptomology and coping strategies. Importantly, the present study also attempted to examine sex differences, which can be especially difficult due to the traditionally smaller representation of women in policing (United Nations Office on Drugs and Crime, 2023). The following *a priori* hypotheses were developed based on prior literature:

1. Frontline officers will display higher levels of PTSI symptoms (including depression, anxiety, and stress) than tactical officers.
2. Tactical officers will exhibit a differential coping profile compared to frontline officers.
3. Male officers will be more likely to use alcohol to cope and will endorse more maladaptive coping strategies.
4. Female officers will report higher levels of PTSI.

## Methods

### Participants

The sample used in the current study are drawn from original data collected between 2014 and 2018 as part of a programmatic line of research conducted within the Health, Adaptation, Research on Trauma (HART) Laboratory led by the principal investigator (fourth author) and collaborators. The HART Lab Police Database (HLPD) was curated through the implementation of a standardized research protocol, developed and refined over time (see Andersen et al., 2024), and applied across multiple study designs in Canada and Finland to examine police officer responses to occupational stressors and to evaluate psychophysiological interventions targeting resilience, health, and performance. Prior publications from HLPD have addressed distinct research questions (e.g., Andersen et al., 2015, 2018; Andersen and Gustafsberg, 2016; Chan and Andersen, 2020; Chan et al., 2022; Di Nota et al., 2021) but no prior research has examined the totality of participants and hypotheses proposed in this study. The HLPD is modeled after a similar PI led, longitudinal police database (BCOPS, Hartley et al., 2011). Additional details on HLPD study timelines, data curation, and publications are provided in the Supplementary (see Table 1).

**Table 1:** Demographic characteristics of frontline and tactical officers.

Demographic variable		Frontline	Tactical
Age	Mean (SD)	32.46 (5.82)	33.96 (5.42)
Sex	Male (%)	101 (81.5%)	53 (98.1%)
	Female (%)	23 (18.5%)	1 (1.9%)
Race	White (%)	88 (71%)	53 (100%)
	African (%)	5 (4%)	0 (0%)
	Indigenous (%)	2 (1.6%)	0 (0%)
	Hispanic (%)	2 (1.6%)	0 (0%)
	South Asian (%)	5 (4%)	0 (0%)
	East Asian (%)	4 (3.2%)	0 (0%)
	Asian American (%)	3 (2.4%)	0 (0%)
	Middle Eastern (%)	1 (0.8%)	0 (0%)
	Multiracial (%)	3 (2.4%)	0 (0%)
	Other (%)	11 (8.9%)	0 (0%)
Years of experience	Mean (SD)	6.99 (5.09)	7.63 (4.17)
Years of experience (current role)	Mean (SD)	7.02 (5.63)	5.42 (4.16)
Country of employment	Canada (%)	124 (100%)	10 (18.5%)
	Finland (%)	0 (0%)	44 (81.5%)

Total sample calculations based on the data in this table may vary from the reported total sample size in the methods section due to participants who did not provide their demographic information.

Inclusion criteria were limited to active-duty police officers aged 18 years and older who were deemed fit for duty (i.e., met agency health requirements for active service and were not on leave). Police administrators and civilian police personnel were excluded from participation. Officers volunteered to participate, and recruitment occurred in collaboration with police agency schedules. All HLPD data collection has been approved by the Social Sciences and Humanities Research Ethics Board (SSHREB) at the University of Toronto and associated police service ethics committees as required according to the Declaration of Helsinki. Due to variable research objectives and some missing/incomplete data on key study variables, the size of the samples in this paper differs from the original sample size from which they are drawn (total sample, *n* = 178; see Supplementary file). Listwise deletion was applied on an analysis-by-analysis basis, such that participants were included in each analysis only if they had complete data for the variables of interest. The two groups of interest are tactical (*n* = 54) and frontline officers (*n* = 124). The tactical officer group consisted of officers from Finland (*n* = 44) and Canada (*n* = 10); police roles and training are comparable across Western contexts. All participants in the frontline officer group were Canadian. About 13% of the current sample are female officers. At the time of data collection, the percentage of female officers in Canada ranged from 13.22 to 18.93%, therefore, the sex distribution in this study was representative of police demographics in Canada at the time of data collection (Statistics Canada, 2024). Both groups were comparable on other relevant demographics including age (mean tactical officer age = 34, mean frontline officer age = 32.5) and years of experience on the police force (mean tactical officer experience = 7.63, mean frontline officer experience = 6.99).

### Materials

The study measures were selected based on validated psychosocial measures commonly used in police research at the time of study inception (see McCreary and Thompson, 2006) and to facilitate comparability between research laboratories. All surveys were collected as part of the baseline demographic and psychosocial assessment component of each study within the HLPD. Surveys were administered immediately prior to the research intervention (e.g., within one week) to minimize potential bias arising from participants’ subsequent study experiences and intervention effects.

### Coping strategies

Coping Strategies were measured through the self-report questionnaire Brief ‘Coping Orientation to Problems Experienced’ (Brief COPE; Carver, 1997). The Brief-COPE includes 14 scales each representing a different coping strategy; this instrument has been previously validated in police samples (including North America and Europe) showing strong psychometric properties (Cronbach’s alpha = 0.85 and test–retest reliability = 0.71; Teles et al., 2024). Each scale is represented by two questions scored on a 4-point Likert scale. Thus, the total score for each scale ranges from 2 to 8. In line with previous literature (Myer et al., 2001; Sirois and Kitner, 2015) and the context of police research in which coping strategies are conceptualized as adaptive or maladaptive, the present study began by analyzing the data using this dichotomy. Adaptive strategies included active coping, planning, use of emotional support, use of instrumental support, positive reframing, acceptance, religion, and humor. Maladaptive strategies included venting, denial, substance use, behavioral disengagement, self-distraction, and self-blame. A final “adaptive” and “maladaptive” score was calculated by adding up the scores for each of the defined strategies into a composite score and taking the average (Meyer, 2001; Sirois and Kitner, 2015). However, due to examinations into the psychometric properties of the Brief-COPE (Rodrigues et al., 2022), and the acknowledgement that broad composite scores tend to reduce interpretability, final interpretation of scores was done at the individual scale level.

### Alcohol use

Alcohol use was measured using the Alcohol Use Disorders Identification Test (AUDIT; Saunders et al., 1993). This is a self-report inventory, divided into three subscales: consumption, dependence, and alcohol related problems. A total score is calculated for each individual based on the sum of the subscales; a total score from 0 to 7 is considered low risk, 8 to 15 is considered risky/hazardous, 16 to 19 is considered high risk/harmful level, and 20 or higher is considered high risk/almost certainly dependent. This tool has previously been validated for use with police (Cronbach’s alpha = 0.81; Davey et al., 2000a, 2000b).

### Post traumatic stress injury

Post Traumatic Stress Injury (PTSI) symptoms were quantified using the following inventories: the Post Traumatic Stress Disorder Checklist Civilian (PCL-C; Lang and Stein, 2005), the Depression Anxiety Stress Scale (DASS42; Lovibond and Lovibond, 1995), and the Hospital Anxiety and Depression Scale (HADS; Bjelland et al., 2002). The PCL-C is a 17-item inventory that specifically quantifies PTSD symptomology experienced in the past month, with higher scores indicating higher symptomology. The questions correspond to the DSM-IV symptoms of PTSD and are presented as 5-point Likert scales. The PCL was developed with combat veterans (Weathers et al., 1993) but has since been used in a sample of police (Hartley et al., 2013). The DASS-42 consists of 42 items and is divided into three subscales: depression, anxiety, and stress. The items are presented as 4-point Likert scales and measure the degree to which participants are experiencing each symptom in the past 7 days. Only the depression and anxiety subscales (14 items each) were considered in the current study. The DASS-42 has been validated with Canadian clinical and community samples (Cronbach’s alpha of 0.97 and 0.92 for depression and anxiety subscales, respectively; Antony et al., 1998) but not with police specifically. The HADS is a 14-item self-report scale which quantifies the severity of depression and anxiety experienced by the individual in the past 7 days; it has been validated in police samples from North America and Europe (Cronbach’s alpha of 0.78 and 0.73 for depression and anxiety subscales, respectively; Teles et al., 2024). Similar to the DASS-42, the HADS presents items on a 4-point scale. In the current data set, depression and anxiety scores were only available for frontline officers from the DASS-42, while depression and anxiety scores were only available for the tactical officers from the HADS. This difference was due to changing methodology between the studies from which the data was pulled. However, the similarity of the scales’ structure and scoring procedure allow them to be directly comparable. This has previously been done by doubling the total HADS score to bring them on the same scale as the DASS-42 (Chan et al., 2022); the same procedure is replicated here. In the final analysis, depression and anxiety scores consist of scores from the DASS-42 and HADS (no participant was given both measures); each construct was tested separately.

Another element of PTSI is occupational and organizational stress, which was quantified using the Police Stress Questionnaire (PSQ; McCreary and Thompson, 2006). This inventor quantifies perceived stress in the past 6 months. Each item is presented as a 7-point Likert scale (1 – ‘no stress’; 4 – moderate; 7 – ‘a lot of stress’). It has 40-items divided into two subscales: organizational stress (20-items – PSQ_org_) and operational stress (20-items - PSQ_op_).

### Statistical analysis

All statistical techniques and data visualizations were carried out on SPSS or R studio (IBM, n.d.; Chang et al., 2017). This project was preregistered on OSF.^1^ Some stated analyses remain consistent with our initial plans while others vary based on data constraints and statistical considerations. Appendix A discusses, provides a detailed breakdown of the analyses conducted for each hypothesis (and how/if they differ from our original analysis plan). Prior to conducting the analyses, assumption checks were done to inform the use of appropriate statistical procedures. For hypothesis 1, the data violated the assumption of multivariate normality; this was checked using the Henze–Zirkler test (*p* < 0.001; Henze and Zirkler, 1990). Therefore, the analysis shifted toward a non-parametric version of MANOVA, specifically permutational MANOVA, and assumptions were checked again. The data satisfied the homogeneity of multivariate dispersions assumption (*p* = 0.28); this was assessed based on a Gower dissimilarity matrix (Anderson, 2006; Gower, 1971) computed using the cluster package in R (Maechler et al., 2025). Frontline and tactical officers were compared on their BRIEF COPE composite scores for maladaptive and adaptive coping strategies as well as each individual coping strategy. AUDIT scores were also analyzed under this hypothesis as a measure of alcohol use, consequently, these scores were included in the permutational MANOVA. However, AUDIT scores were not factored into either composite score, as they are psychometrically distinct from the Brief Cope. *Post hoc* Wilcoxon rank sum tests were conducted to pinpoint the specific differences between groups across the omnibus test; *p* values were adjusted using the false discovery rate (FDR) method in R. For hypothesis 2, the assumption of normality was violated for operational stress (*p* < 0.05 for both operational and organizational), depression/anxiety (*p* < 0.05), and PTSD (*p* < 0.05) using the Shapiro–Wilks test and visual inspection. Therefore, hypothesis 2 was tested using Wilcoxon rank sum tests for each dimension of PTSI. A *post hoc* FDR correction was applied to account for multiple tests. For hypothesis 3, the assumption of multivariate normality was violated (Henze–Zirkler test; *p* < 0.001), therefore permutational MANOVA was used to assess the difference in coping between the sexes (no post hoc testing was needed due to a lack of significance). Similar to above, the data satisfied the assumption of homogeneity of multivariate dispersions (*p* = 0.39) based on a Gower dissimilarity matrix (Anderson, 2006; Gower, 1971) computed using the cluster package in R (Maechler et al., 2025) Finally, for hypothesis 4, the assumption of normality was violated for operational stress (*p* < 0.05 for both operational and organizational), depression/anxiety (*p* < 0.05), and PTSD (*p* < 0.05) using the Shapiro–Wilks test and visual inspection. Hypothesis 4 was therefore tested using Wilcoxon rank sum tests to assess the difference in PTSI symptoms between the sexes. It is important to note that tactical and frontline officers were collapsed into the same groups for all sex-based analyses given the small number of females. Effect sizes (e.g., *r*, *η_p_*^2^) and confidence intervals were used to contextualize the results where applicable.

## Results

### Sample characteristics

See Table 1 for the demographic breakdown of each group. Both groups of interest were comparable in terms of their age, years of experience, and their years of experience in their current role. The current sample is predominantly male, however, the sex distribution of the current sample was comparable with general Canadian officer demographics (Statistics Canada, 2024) at the time of data collection. Frontline officers were majority white but also varied in terms of other races including African, Hispanic, Asian, Middle Eastern, Indigenous, Multiracial, and other. The entire tactical sample identified as white.

### PTSI by police role

**Figure 1:**
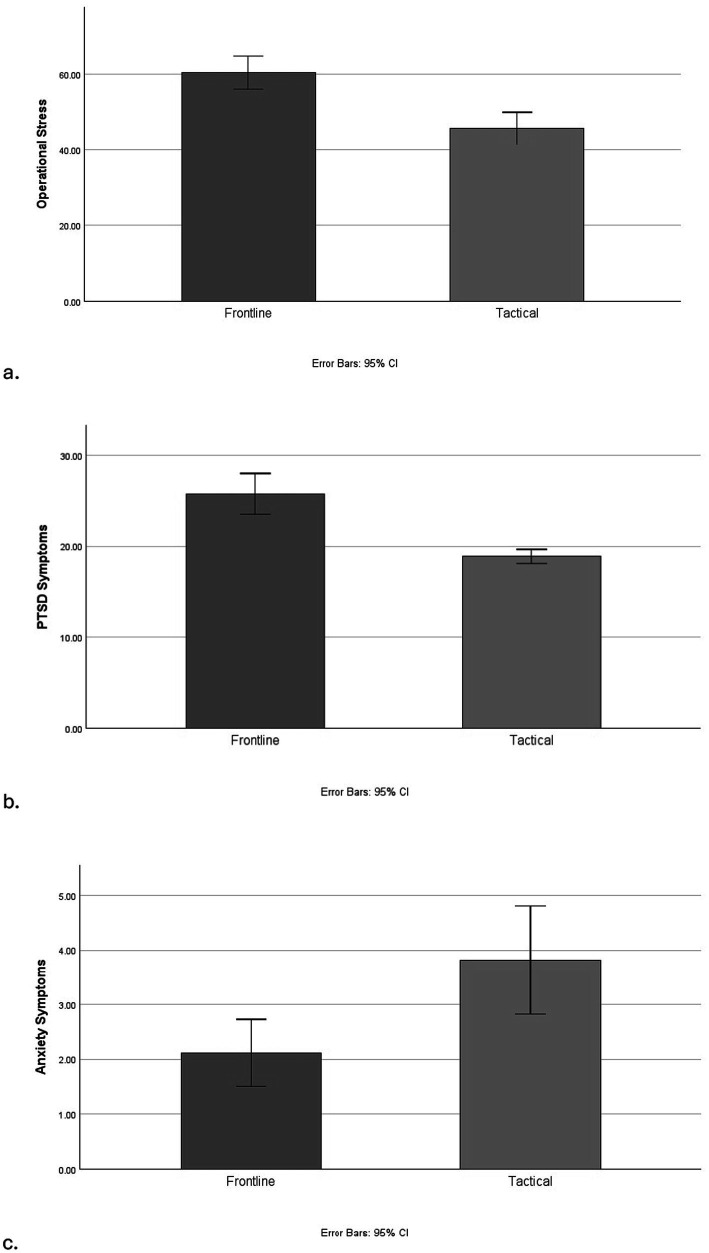
**(a)** Is a bar graph comparing the difference in mean scores of operational stress scores between frontline and tactical officers (error bars represent 95% confidence intervals). **(b)** Is a bar graph comparing the difference in mean PCL-C scores between frontline and tactical officers (error bars represent 95% confidence intervals). **(c)** Is a bar graph comparing the difference in mean anxiety scores (DASS-42 and HADS) between frontline and tactical officers (error bars represent 95% confidence intervals).

There was no significant difference in depression (*w* = 3458.00, *p*_FDR_ > 0.05, *r* = 0.13) between frontline and tactical police. There was a significant difference in anxiety scores (*w* = 2017.50, *p*_FDR_ < 0.001, *r* = 0.28) with a small effect size, such that tactical police reported higher anxiety scores. Frontline officers reported significantly higher levels of PTSD symptoms than tactical officers (*w* = 2540.50, *p*_FDR_ < 0.001, *r* = 0.68) with a large effect. Police groups did not significantly differ in their levels of organizational stress (2601.50, *p*_FDR_ > 0.05, *r* = 0.08), however, frontline police reported significantly higher levels of operational stress than tactical police (*w* = 3283.00, *p*_FDR_ < 0.001, *r* = 0.36); a medium effect was observed (Figure 1).

### Coping strategies by police role

Table 2 outlines scores for frontline and tactical officers on measures of coping derived from the Brief Cope. Permutational MANOVA revealed a significant difference between frontline and tactical officers in terms of their coping strategies with a medium effect (*F*(1, 166) = 15.11, *p* < 0.001, *η_p_*^2^ = 0.083). *Post hoc* testing revealed that tactical officers scored higher on both the adaptive (*W* = 1,777.5, *p*_FDR_ < 0.001, *r* = 0.34) and maladaptive coping (*W* = 2,148, *p*_FDR_ < 0.01, *r* = 0.25) composite scores. Tactical officers scored higher on the following specific coping strategies; acceptance (*W* = 1,583.5, *p*_FDR_ < 0.001, *r* = 0.40), emotional support (*W* = 1,869.5, *p*_FDR_ < 0.001, *r* = 0.32), instrumental support (*W* = 2,102, *p*_FDR_ < 0.001, *r* = 0.26), venting (*W* = 1,512, *p*_FDR_ < 0.001, *r* = 0.42), positive reframing (*W* = 1,879, *p*_FDR_ < 0.001, *r* = 0.32), planning (*W* = 2,148, *p*_FDR_ < 0.01, *r* = 0.25), humor (*W* = 2,114.5, *p*_FDR_ < 0.001, *r* = 0.26), and self-blame (*W* = 2,024.5, *p*_FDR_ < 0.001, *r* = 0.29). AUDIT scores were also analyzed under this hypothesis. Although initially significant (*W* = 2,623.5, *p* < 0.05), alcohol consumption did not remain significant after FDR correction (*W* = 2,623.5, *p*FDR > 0.05, *r* = 0.15); a small effect was identified.

**Table 2:** Brief cope scores, confidence intervals, significance, and effect sizes for frontline and tactical officers.

Coping variables		Frontline officers	Tactical officers
Adaptive coping composite	Mean score	33.3	39.6
	Mean 95 CI	[31.5, 35.1]	[37.4, 41.9]
	Significant?	Yes	
	Effect size (*r*)	0.34	
Maladaptive coping composite	Mean score	17.2	19.2
	Mean 95 CI	[16.5, 17.9]	[18.1, 20.3]
	Significant?	Yes	
	Effect size (*r*)	0.25	
Acceptance*	Mean score	4.66	6.19
	Mean 95 CI	[4.33, 4.99]	[5.76, 6.61]
	Significant?	Yes	
	Effect size (*r*)	0.40	
Active coping*	Mean score	4.82	5.13
	Mean 95 CI	[4.50, 5.13]	[4.67, 5.59]
	Significant?	No	
	Effect size (*r*)	0.10	
Self distraction	Mean score	3.98	3.89
	Mean 95 CI	[3.73, 4.24]	[3.48, 4.30]
	Significant?	No	
	Effect size (*r*)	0.03	
Denial	Mean score	2.21	2.24
	Mean 95 CI	[2.10, 2.32]	[2.07, 2.42]
	Significant?	No	
	Effect size (*r*)	0.05	
Substance	Mean score	2.22	2.26
	Mean 95 CI	[2.10, 2.34]	[2.07, 2.44]
	Significant?	No	
	Effect size (*r*)	0.03	
Emotional support*	Mean score	4.28	5.33
	Mean 95 CI	[3.97, 4.59]	[4.93, 5.74]
	Significant?	Yes	
	Effect size (*r*)	0.32	
Instrumental support*	Mean score	3.93	4.67
	Mean 95 CI	[3.64, 4.22]	[4.31, 5.03]
	Significant?	Yes	
	Effect size (*r*)	0.26	
Behavioral disengagement	Mean score	2.28	2.15
	Mean 95 CI	[2.14, 2.42]	[2.02, 2.27]
	Significant?	No	
	Effect size (*r*)	0.04	
Venting	Mean score	3.29	5.69
	Mean 95 CI	[3.07, 3.51]	[4.26, 5.11]
	Significant?	Yes	
	Effect size (*r*)	0.42	
Positive reframing*	Mean score	4.29	5.35
	Mean 95 CI	[3.98, 4.60]	[4.99, 5.72]
	Significant?	Yes	
	Effect size (*r*)	0.32	
Planning*	Mean score	4.45	5.33
	Mean 95 CI	[4.11, 4.78]	[4.95, 5.72]
	Significant?	Yes	
	Effect size (*r*)	0.25	
Humor*	Mean score	4.26	5.11
	Mean 95 CI	[3.93, 4.59]	[4.70, 5.52]
	Significant?	Yes	
	Effect size (*r*)	0.26	
Religion*	Mean score	2.63	2.5
	Mean 95 CI	[2.39, 2.87]	[2.23, 2.77]
	Significant?	No	
	Effect size (*r*)	0.03	
Self blame	Mean score	3.23	4
	Mean 95 CI	[2.98, 3.48]	[3.64, 4.36]
	Significant?	Yes	
	Effect size (*r*)	0.29	

*denotes coping strategies that are a part of the adaptive composite score.

### Sex comparisons

No significant differences were found in coping strategies endorsed between the sexes (*F*(1, 166) = 1.69, *p*FDR > 0.05, *η_p_*^2^ = 0.01). Male and female officers were also assessed for differences in PTSI; no significant differences in reports of depression (*w* = 1,461, *p*FDR > 0.05, *r* = 0.02), anxiety (*w* = 1,674.50, *p*FDR > 0.05, *r* = 0.06), PTSD symptomology (*w* = 5424.50, *p*_FDR_ > 0.05, *r* = 0.02), or operational/organizational stress (*w* = 1,514, *p*FDR > 0.05, *r* = 0.071; *w* = 1,230.5, *p*FDR > 0.05, *r* = −0.051). Lastly, there was no significant difference in alcohol use between men and women (*w* = 1,800.00, *p*FDR > 0.05, *r* < 0.01).

## Discussion

The present study compared frontline and tactical police officers on post-traumatic stress injury (PTSI) symptomatology and coping strategies to clarify role specific strengths and vulnerabilities. There were no significant differences in alcohol use between groups and no sex based differences were observed. Frontline officers reported higher levels of PTSD symptomatology and operational stress, but lower anxiety scores, whereas tactical officers endorsed a broader and more frequent use of coping strategies.

However, when discussing coping strategies it is important to note that they are not inherently good or bad, but rather have a different effectiveness based on the situation. For example, Bartram and Gardner (2008) note that problem-focused strategies might be unhelpful for an unchangeable situation while emotion-focused strategies could be more effective here. Yet, when the situation is changeable, the opposite might be true. Thus, most coping mechanisms exhibited in the Brief-COPE cannot be seen as necessarily adaptive or maladaptive but depend on the situation for which they were used.

### Frontline officers

Frontline officers reported significantly greater PTSD symptomatology and operational stress than tactical officers, consistent with prior research on operational policing stress exposure (Ricciardelli et al., 2020; Planche et al., 2019). This pattern is notable given that tactical officers are often exposed to more acute and extreme traumatic incidents. One plausible explanation concerns differences in training and preparedness. Tactical officers typically receive extensive, ongoing role specific training, whereas frontline officers often complete only several months of basic training before being expected to manage a wide range of potentially traumatic calls (Towns and Ricciardelli, 2025; Toronto Police Service, n.d.) Insufficient or fear-based training may increase perceived threat, risk of behavioral errors, and reduce perceived coping capacity during stressful and potentially traumatic critical incidents (Harman et al., 2019). Another explanation could be that tactical officers work largely in cohesive units, while frontline workers may work independently or with different officers based on availability; the difference in social structure may also influence stress perception (Planche et al., 2019).

Frontline officers also did not endorse higher use of any coping strategy relative to tactical officers, suggesting a less proactive and less flexible coping profile. Empirical research indicates that coping flexibility, defined as the ability to select coping strategies that align with situational demands, is a critical determinant of psychological distress, particularly under conditions of high stress (Leslie-Miller et al., 2025). When coping responses are poorly matched to perceived stressor controllability, psychological distress is elevated, whereas greater strategy situation fit is associated with lower depressed mood (Leslie-Miller et al., 2025). This framework is especially relevant to policing, where critical incidents often involve high stress combined with limited controllability, suggesting that reduced coping flexibility among frontline officers may contribute to their heightened psychological distress. However, the directionality of this relationship cannot be determined from correlational data, as heightened PTSD symptoms may also impair coping capacity (Weiss et al., 2019). Longitudinal research is required to disentangle these mechanisms.

### Tactical officers

Tactical officers reported significantly greater use of multiple coping strategies, including acceptance, emotional and instrumental support, planning, positive reframing, humor, religion, venting, and self-blame. This broader coping repertoire may reflect organizational and environmental differences inherent in tactical and frontline roles rather than individual differences alone. As mentioned previously, tactical officers are required to work in teams and must be able to trust fellow officers with their life (Finnish Police, n.d.; Toronto Police Service, n.d.), thus team cohesion may also contribute to the finding that operational stress was lower. However, team cohesion was not measured here, therefore this explanation remains speculative. However, literature exists to support this explanation. Specifically, a study by Bekesiene et al. (2022) examined group cohesion in a sample of Lithuanian military conscripts and demonstrated that increased cohesion was significantly associated with a reduction of perceived stress experienced by the soldiers. Indeed, strong team solidarity may make it possible to obtain social support from people who understand what you are going through. This presumably provides tactical officers with increased opportunities to seek understanding and comfort from others (emotional support), solicit advice and practical support (instrumental support), and express their frustrations or anger (venting). Other research indicates that social engagement with trusted, similar others, fosters psychological safety and is associated with positive co-regulatory physiological processes that support health and well-being (Porges, 2022). However, associations between team cohesion and co-regulatory physiology remain largely unexplored in police populations, are speculative in the current study, warranting future research. Another explanation for reduced operational stress among tactical police could be a greater sense of preparedness. As discussed previously, these officers undergo a rigorous selection process to serve on specialized units while also maintaining robust physical standards (Towns and Ricciardelli, 2025; Toronto Police Service, n.d.). This also remains speculative, as the current study did not measure preparedness (or a similar construct).

As mentioned above, tactical officers in the current study reported significantly greater use of a broad range of coping strategies, including both adaptive (e.g., acceptance, planning, positive reframing, humor) and less adaptive responses (e.g., venting, self-blame), while also endorsing higher anxiety but lower PTSI symptoms. This pattern may reflect heterogeneity in underlying response profiles rather than a uniform stress response. Consistent with this interpretation, Garbarino et al. (2012) identified distinct personality-based subgroups among tactical officers, including a more resilient group characterized by effective stress regulation and a less resilient group with profiles comparable to the general population. Applied to the present findings, elevated anxiety may coexist with active coping processes that limit progression to PTSI in some officers, while variability in coping strategies suggests differences in underlying personality and stress regulation. Personality factors may contribute to these patterns, as prior work indicates that personality influences coping responses (Jacobs and Carver, 2020), and evidence from Dutch special forces shows lower neuroticism and higher conscientiousness relative to comparison groups (Huijzer et al., 2022). However, the current sample size, and lack of broad personality measures precludes analysis testing distinct coping and personality profiles. Future research, including qualitative approaches, is warranted to examine personality differences between frontline and tactical officers and to clarify mechanisms underlying coping profiles and differential vulnerability to PTSI.

Tactical officers also reported higher self-blame, which may reflect group norms or conformity pressures rather than adaptive coping. Elevated self-blame may be indicative of accountability cultures or internalized performance standards (Costanzo, 1970). Future research examining team dynamics within tactical units may clarify whether this pattern reflects cohesion, conformity, or maladaptive internalization.

### Alcohol use

AUDIT scores did not differ by police role or sex, consistent with the Brief COPE substance use subscale. Approximately 12% of the sample met criteria for risky drinking which might be considered low compared to other studies in different cultural contexts (Irizar et al., 2021; Houdmont and Jachens, 2022; Ndumwa et al., 2023; Ovuga and Madrama, 2006). Interestingly, our sample closely mirrors general population trends in Canada, according to government data, roughly 11.4% of adults meet the AUDIT criteria for risky drinking (Government of Canada, 2025). In Finland, this figure is greater, with roughly 28% of adults meeting the AUDIT criteria for risky drinking (Finnish Institute for Health and Welfare, 2024). Of course, it is not surprising that the current sample mirrors Canadian trends more closely because the majority of officers included here are Canadian. Few studies have examined differences in alcohol consumption between police and the general population. However, one study examining protective service personnel (including police) found no difference in AUDIT scores with other occupations (Weir et al., 2012). The finding that alcohol consumption patterns do not differ between police roles aligns with the narrow literature that exists on this topic. Carleton et al. (2018) examined alcohol use via the AUDIT between many PSP categories (but did not examine sex differences in isolation). They found no difference in AUDIT scores between municipal/provincial police and Royal Canadian Mounted Police (Carleton et al., 2018). In another study, Norwegian police were found to have similar patterns of alcohol use as ambulance personnel (using the AUDIT; Sterud et al., 2007).

### Sex differences

Investigations of sex differences among police have, at times, yielded different conclusions. For example, Carleton et al. (2018) reported that among municipal/provincial police, women were more likely than men to report mental health symptoms. However, in a more recent investigation, Carleton et al. (2024) reported that, in a sample of Royal Canadian Mounted Police, women were less likely to screen positive for PTSD and alcohol use disorder. The authors suggested that socialization and openness to endorsing mental health symptoms may contribute to sex differences in mental health screening. Indeed, expression of and help-seeking for emotional concerns may be more socially accepted among women (Mankus et al., 2016). Higher rates of service utilization among female officers may reflect sex differences in perceived and enacted stigma. Women may experience greater social acceptance of emotional disclosure and help-seeking, whereas men may be less likely to seek support due to concerns about negative evaluation, including peer ridicule and stigmatization within male-dominated occupational cultures. Other scholars have called for research that moves beyond descriptive symptom checklists to examine underlying mechanisms that may drive observed differences, such as empathy and stress regulation capacity (Beagley et al., 2018). Several studies have investigated barriers to accessing mental health care among police (Newell et al., 2021; Drew and Martin., 2021), yet studies have not reported results stratified by sex, potentially due to lower numbers of female officers. The lack of knowledge underscores the importance of examining sex differences in future studies.

Sex differences in alcohol use also appear to be nuanced. Our findings align with Ballenger et al. (2011) who also found no sex differences in alcohol use among American police officers. However, our results diverge with other studies that have found sex differences (Houdmont and Jachens, 2022; Ménard and Arter, 2014; Davey et al., 2000a, 2000b). Similarly to Ballenger et al. (2011), we posit that because policing is a male-dominate environment, drinking might be more normalized, and it is possible that female officers become acculturated to this environment. Women may also feel a greater pressure to prove their competence by engaging in behaviors that may lead to acceptance by their male colleagues (i.e., alcohol consumption). One study by Gutschmidt and Vera (2022) demonstrated a link between conservative male culture and maladaptive coping strategies (such as drinking alcohol) in police, giving credence to the idea that female police drinking may be influenced by male culture. This remains speculative and requires further examination.

### Methodological considerations and limitations

The results of the current study may not generalize to all police. Although these samples share broadly similar Westernized cultural and demographic contexts, cross-national differences in policing practices and population characteristics cannot be fully excluded. However, Finnish tactical units are comparable to their Canadian counterparts in key operational domains, including selection processes, physical fitness requirements, training demands, and exposure to high-risk incidents (Finnish Police, n.d.; Toronto Police Service, n.d.). Notably, the female subsample was small and thus limited statistical power, which may explain the lack of sex differences. Furthermore, this small subsample prevented us from conducting additional analyses stratified by sex (i.e., comparing tactical females with frontline females). While there are very few female police participants, the sex distribution in the samples is proportionate to and reflects the demographics of the male-dominated profession of policing in at the time of data collection (Statistics Canada, 2024; Andersen et al., 2018).

Similar to other studies utilizing psychosocial questionnaires, we acknowledge the possibility of response bias (i.e., social desirability) when using self-report measures. However, given that the psychosocial measures were completed before the study began and completed without the researchers present, there is not a strong reason to believe that any systematic bias was present or that it influenced one group more than the other. However, completing self-report measures may have led to artificially lower reporting of PTSI symptoms and alcohol use in both groups. Finally, all the officers included in this study volunteered to participate, which introduces the possibility of self-selection bias. Officers who are more health conscious or curious about research may have volunteered to participate at higher rates. However, it is also possible that eligible participants interested in volunteering were not able to due to work schedules that were beyond their control (see Supplementary file).

Although the composition of the sample introduces certain limitations and is based on correlational data, applied research remains essential to strengthening psychological science by situating findings in real world contexts, improving external validity, and capturing forms of complexity that are difficult to observe in laboratory settings (Andersen, 2025). A further consideration is that our interpretation of coping strategies was guided by examination of individual Brief COPE subscales rather than adaptive versus maladaptive composites. Detailed justification and psychometric considerations are provided in the Supplementary file.

### Future directions

In conclusion, meaningful differences were found between police roles pertaining to their experience of PTSI symptomology and use of coping strategies. These findings underscore the importance of examining occupational subgroups within policing as they may inform differential treatment approaches. Future research may inform targeted interventions, including enhanced training requirements for frontline officers, organizational practices that promote team cohesion and positive physiological co-regulation (McCraty and Zayas, 2014; Porges, 2022), and expanded mental health supports aimed at strengthening adaptive coping capacity and flexibility (Leslie-Miller et al., 2025).

## Data Availability

The datasets presented in this article are not readily available because given the extremely confidential nature of data assessing mental health of emergency workers, the data is not publicly available. Further questions about access to the data can be directed to the corresponding author. Requests to access the datasets should be directed to judith.andersen@utoronto.ca.

## Footnotes

1
https://doi.org/10.17605/OSF.IO/BU923
