# Supplementary material for: Coping strategies and post traumatic stress injury symptoms differ between tactical and frontline police officers: implications for health and public safety
Source: Front Psychol. 2026 May 28;17:1799457. doi: 10.3389/fpsyg.2026.1799457 (PMC13254071; doi:10.3389/fpsyg.2026.1799457)
Supplement: Supplementary file 1 [file Data_sheet_1.pdf]

Supplementary

Rationale for Analysis Plan

Hypothesis 1 was tested using permutational MANOVA. For this test, frontline and tactical officers were compared on their BRIEF COPE composite scores for maladaptive and adaptive coping strategies as well as each individual coping strategy. Post hoc Wilcoxon rank sum tests were conducted to pinpoint the specific differences between groups. P values were than adjusted using the false discovery rate method in R. Our initial plan for hypothesis 1 (individual t-tests or nonparametric equivalent) was fine for an analysis of just an adaptive and maladaptive composite. However, due to reasoning discussed in the paper (deeper dive into the factor structure of the Brief COPE and the lack of interpretability offered by the composite scores) we decided to analyze the coping strategies individually. Sticking to individual t-tests would have greatly increased our risk for type 1 error, therefore we conducted the permutational MANAOVA to increase the power/validity of the analysis. Hypothesis 2 was tested using Wilcoxon rank sum tests for each dimension of PTSI (operational stress, depression/anxiety, and PTSD symptoms). Our initial plan was to evaluate these differences using a MANOVA, however, missing data would have significantly reduced the sample (i.e. for some measures tactical officer data was missing and on others frontline officer data was missing). Therefore, we elected to conduct separate tests with a post hoc false discovery rate correction in R. Hypotheses 3 was tested using a permutational MANOVA. Post hoc Wilcoxon rank sum tests were used to identify the specific differences between groups. Similar to hypothesis 1, we pivoted from the initial plan to combat against type 1 error. Hypothesis 4 was tested using Wilcoxon rank sum tests (PTSI sex differences). Finally, the OSF also included a hypothesis about the possibility of social support (IESL) moderating the relationship between alcohol use (AUDIT) and maladaptive coping. This test was not conducted because it was later discovered that there was no social support data for the tactical officers.

Rationale for Analyzing Coping Strategies

The present analysis initially followed a dichotomous structure (adaptive or maladaptive) often presented in police literature and other studies using the Brief COPE (Alosaimi et al., 2018; Mohanraj et al., 2015; Meyer 2001). However, examinations into the psychometric properties of the Brief Cope strongly suggests that a two-factor structure may not be valid (Rodrigues et al., 2022) and other researchers have suggested alternate factor structures (Cooper et al., 2008). Although there are a number of factor structures proposed in the literature (Cooper et al., 2008; Richards et al., 2022), the 14-factor structure (i.e. examining all coping strategies separately) has consistently been supported (Rodrigues et al., 2022); the prudent approach is to interpret the data in accordance with the sample being examined (i.e. consider relevant sample characteristics) rather than collapsing the data into potentially heterogeneous composite scores. Therefore, taking a step back from the maladaptive/adaptive dichotomy, the data appears to indicate that tactical officers are more proactive than frontline officers in their use of coping strategies.

HARTLAB Police Database (HLPD)

The HART Lab Police Database (HLPD) has been developed and led by the Principal Investigator, Judith P. Andersen, PhD since its inception in 2013-2026 (to present day), in collaboration with successive teams of students and research partners. Dr. Andersen, a health psychologist and Associate Professor in Psychological and Brain Sciences at the University of Toronto Mississauga, has secured continuous research ethics board approvals at the University of Toronto and federal and provincial funding to support the studies comprising the HLPD. Of note, funders have no access to the data and do not influence publication decisions.

Dr. Andersen has directly overseen all data collection and database management within the HART Lab to ensure adherence to standardized protocols, research ethics approvals and participant confidentiality practices. Ethical oversight is maintained through the Social Sciences and Humanities Research Ethics Board at the University of Toronto (SSHREB), in coordination with various ethics committees at participating police organizations as required and in line with the guidelines of the Declaration of Helsinki. No research data is shared with police agencies or stored at any other location than the HART Lab. All data is de-identified and stored on an internal server in locked offices at the University of Toronto and accessible only by approved members of the research team. Where allocation to control/experimental group or trainer's ratings are considered, all police evaluators were blinded to participant group allocation. To protect the confidentiality of participants providing sensitive information, the HLPD is not publicly available. Inquiries regarding ethics approval for data access can be directed to Dr. Andersen.

Participant recruitment and attrition

As with any field research, there are factors that limit participation among eligible individuals. In policing, operational demands take precedence, requiring officers to remain available for shift work, with schedules that can change rapidly in response to real-world needs. This context can restrict an officer's ability to participate in or complete a study. Because scheduling is determined independently of study recruitment and assignment, and reflects a systemic feature across police services, the authors posit that it is unlikely to introduce systematic bias in participation or attrition rates among eligible officers even though it inhibits researchers from calculating the true rate of eligible participants that did not participate (because we as researchers are not provided the information from the police service) or who were lost to attrition. Although this constraint is inherent to field research, it also enhances ecological validity. Data collected under real-world conditions provide contextually grounded insights that complement findings from controlled laboratory settings, despite the logistical limitations associated with this population (Andersen, 2025).

Tactical Officers: All data from tactical officers was collected within 6 months in 2014, limiting temporal differences.
Canadian Tactical: A team of 10 officers were invited to participate in the first HART Lab pilot protocol study and completed the baseline measures (including the psychosocial surveys analyzed in the present study). Of the total, 8 completed varying days of physiological measurement over the course of 30 days of observation as scheduling permitted.

Finland tactical teams: All invited participants in each tactical team from Finland participated (with no more than 2 lost to attrition due to illness). According to our research collaborators in Finland, high participation rates are common given the openness and interest in research in that country and the centralized and accessible location of the data collection (Finnish Police College).

Canadian Frontline officers:
Recruitment: The total pool of active-duty officers at the large urban police service in Ontario, Canada was approximately 750. Study inclusion criteria were any frontline officer who had completed all basic training, were not being supervised by a "coach" officer and were deemed as "fit for duty" according to the service's standards and were present on the days that researchers attended the service to recruit study participants or those who answered study advertisement communications sent by email from the police service (number of emails was not provided). Exclusion criteria were un-sworn police service workers (i.e., civilian staff), officers who were on medical leave or vacation at the time of study recruitment and evaluation, and officers deemed unfit for duty by the police service. The police service did not provide us with an estimate of the number of individuals that met exclusion criteria from the total pool and thus it is not possible to calculate bias in response due to eligibility.
Attrition: Attrition rates were different based on study design (e.g., RCT, Longitudinal) and thus must be evaluated per sample. Please see table 1 below for the publication associated with each study design in order to find out more information about attrition per study.

Present Study
Data in the current study are drawn from HLPD police samples collected between 2014 and 2018 in Canada and Finland, all of which employed the consistent standardized baseline psychosocial questionnaires previously used and validated with police samples that administered to participants after signing informed consent and immediately prior to baseline data collection to avoid biasing responses by exposure to any of the research intervention protocol. The only variation in measures was the replacement of the HADS (tactical officers) with the DASS (frontline officers) although scores have been standardized in order to be examined on the same scale.

Table 1 includes all data collected between 2014 and 2018, corresponding to the dataset analyzed in this paper; it does not represent the full HLPD dataset (2014–2025). Specific study dates (month and day) are not reported to reduce the risk of identifying individual officers, particularly given the small sample sizes, and to preserve participant confidentiality. Requests for additional detail regarding the full HLPD or specific study dates may be directed to the Principal Investigator.

Also of note, the extensive number of measures collected in each study and the varying number of study days was on purpose in order to derive a more comprehensive health and performance profile on police in Canada and Europe but by that very issue it precludes any one paper from containing all possible variables and outcomes. Similar database curation has been done by another large scale laboratory on police stress and health. Specifically, the Buffalo Cardio-Metabolic Occupational Police Stress (BCOPS) study run by PI John Violanti (Hartley et al., 2011; Charles et al., 2011). The BCOPS study was originally proposed as a cross-sectional pilot (n=100), 2004-2006, has grown in size to over 400 police officers and refined measurement scales and study designs since its inception to present day. Numerous collaborative studies police health and functioning associated with stress continue to the present time and serve as a model for the HLPD.

Hartley TA, Burchfiel CM, Fekedulegn D, Andrew ME, Violanti JM. Health disparities in police officers: comparisons to the U.S. general population. Int J Emerg Ment Health 2011; 13:211–22

Charles LE, Violanti JM, Gu JK, Fekedulegn D, Andrew ME, Burchfiel CM. Sleep duration and biomarkers of metabolic function among police officers. J Occup Environ Med 2011; 53:831–837.

HLPD References

Andersen, J.P., *Di Nota, P.M., Alavi, N., Anderson, G.S., Bennell, C., McGregor, C., Ricciardelli, R., *Scott, S.C., Shipley, P. & Vincent, M. (2023). Autonomic modulation training protocol: A biological approach to building resilience and wellness capacity among police exposed to post-traumatic stress injuries. JMIR Research Protocols. http://dx.doi.org/10.2196/33492

Marlette, H., Di Nota, P. M., & Andersen, J. P. (2025). Physiological stress differentially impacts cognitive performance during and memory following simulated police encounters with persons experiencing a mental health crisis. *Frontiers in Psychology*, 16, Article 1549752. https://doi.org/10.3389/fpsyg.2025.1549752

*Di Nota, P.M., *Scott, S.C., Huhta, J.-M., Gustafsberg, H., & Andersen, J.P. (2024). Physiological responses to organizational stressors among police managers. *Journal of Applied Psychophysiology and Biofeedback*, DOI: https://doi.org/10.1007/s10484-023-09613-2

*Di Nota, P. M., Huhta, J. M., Boychuk, E.C. & Andersen, J. P. (2023) Police lethal force errors and stress physiology during video and live evaluation simulations, *Police Practice and Research*, DOI: 10.1080/15614263.2023.2237624 IF 1.8

*Chan, J. F., *Di Nota, P. M., *Planche, K., Borthakur, D., Andersen, J. P. (2022). Associations between police lethal force errors, measures of diurnal and reactive cortisol, and mental health. *Psychoneuroendocrinology*. 142(2022). Doi: https://doi.org/10.1016/j.psyneuen.2022.105789

Andersen, J. P., *Di Nota, P. M., Boychuk, E. C., Schimmack, U., & Collins, P. I. (2021). Racial Bias and Lethal Force Errors Among Canadian Police Officers. *Canadian Journal of Behavioural Science* / Revue canadienne des sciences du comportement. http://dx.doi.org/10.1037/cbs0000296 **was republished in 2023 as well*.

*Di Nota PM, Arpaia J, Boychuk EC, Collins PI and Andersen JP. (2021) Testing the Efficacy of a 1-Day Police Decision-Making and Autonomic Modulation Intervention: A Quasi-Random Pragmatic Trial. *Front. Psychol.* 12:719046. doi: 10.3389/fpsyg.2021.719046

*Chan, J & Andersen, J. P. (2020). The Influence of Organizational Stress on Reported Depressive Symptoms Among Police. *Occupational Medicine*. 70, 496-502. doi:10.1093/occmed/kqaa141

*Chan, J. F., & Andersen, J. P. (2020). Physiological Stress Responses Associated with High-Risk Occupational Duties. *Occupational Health*. DOI: http://dx.doi.org/10.5772/intechopen.93943

Planche, K., *Chan, J., *Di Nota, PD., Beston, B., Boychuk, E., Collins, PI., & Andersen, JP. (2019). Diurnal cortisol variation according to high risk occupational specialty within police: comparisons between frontline, tactical officers, and the general population.** *Journal of Occupational and Environmental Medicine.* doi: 10.1097/JOM.0000000000001591

Andersen, J. P., *Di Nota, P., Beston, B., Boychuk, E. C., Gustafsberg, H., Poplawski, S., & Arpaia, J. (2018). Reducing lethal force errors by modulating police physiology. *Journal of Occupational and Environmental Medicine:* 6(10). Pg. 867-874. doi: 10.1097/JOM.0000000000001401

Andersen, J. P., & Gustafsberg, H. (2016). A Training Method to Improve Police Use of Force Decision Making: A Randomized Controlled Trial. *Journal of Police Emergency Response*. Pg 1-13. SAGE Open. doi: 10.1177/2158244016638708.

Andersen J.P., Papazoglou K, Collins P. (2016) Reducing Robust Health-Relevant Cardiovascular Stress

Responses Among Active-Duty Special Forces Police. *Gen Med*. 4:225. doi:10.4172/2327 5146.1000225

Andersen, J. P., Pitel, M., Weerasinghe, A., & Papazoglou, K. (2016). Highly realistic scenario based training simulates the psychophysiology of real world use of force encounters: Implications for improved police Officer Performance. *Journal of Law Enforcement. Open Access*: ISSN: 2161-02331, 5(4), 1-13

Andersen, J. P., Dorai, M., Papazoglou, K., & Arnetz, B. B. (2016). Diurnal and reactivity measures of cortisol in response to intensive resilience and tactical training among special forces police. *Journal of Occupational and Environmental Medicine*. 58(7) e242-248.
